# Reliability Evaluation of Fluorescence In Situ Hybridization (FISH) and G-Banding on Bone Marrow and Peripheral Blood Cells in Chronic Myelogenous Leukemia Patients

**DOI:** 10.22074/cellj.2015.525

**Published:** 2015-04-08

**Authors:** Soheila Manaflouyan Khajehmarjany, Seyed Ali Rahmani, Seyed Hadi Chavoshi, Ali Esfahani, Ali Akbar Movassaghpour Akbari

**Affiliations:** 1 Department of Cellular and Molecular Biology, Islamic Azad University, Ahar Branch, East Azerbaijan, Iran; 2 Dr. Rahmani Medical Genetic Lab, Tabriz, East Azerbaijan, Iran; 3 Department of Hematology and Oncology Research Center, Tabriz University of Medical Sciences, Tabriz, East Azerbaijan, Iran; 4 Emam Reza Hospital, Tabriz, East Azerbaijan, Iran

**Keywords:** CML, FISH, Philadelphia Chromosome, Cytogenetic

## Abstract

Chronic myeloid leukemia (CML) is a myeloproliferative disease. The cytogenetic hallmark of CML is Philadelphia (Ph) chromosome. This study aimed to diagnose suspected CML patients, to monitor CML patients under therapy using cytogenetic and fluorescence in situ hybridization (FISH) techniques to analyze their bone marrow (BM) and peripheral blood (PB) samples, and finally to compare their obtained results for both specimens. This study was conducted during one-year period (2012-2013). The participants were recruited from the Hematology and Oncology Clinic of Shahid Gazi (Emam Reza) Hospital of Tabriz University of Medical Sciences, Tabriz, East Azerbaijan Province, Iran. We analyzed 90 samples from 60 suspected CML patients (30 BM and 60 PB samples). All samples were analyzed using G-banding, 5 samples using dual fusion FISH (DF-FISH) probes, as well as 30 samples using both FISH and G-banding. Among the 90 analyzed samples of 60 patients, 25 (41.66%) were Ph+ using karyotyping, whereas five cases were not analyzable, so FISH was applied and the results confirmed that only two individuals were *BCR-ABL*+. In the comparison between 25 BM and 25 PB samples using karyotyping, 15 (60%) and 10 (40%) were ph+, respectively. The comparison of FISH and karyotyping on 30 samples showed that 9 (30%) and 8 (26.66%) were Ph+, respectively, and only 18.18% of Ph+ patients showed atypical patterns. In the comparison between BM-cytogenetic and PB- interphase-FISH (I-FISH), BM-cytogenetic was more reliable than PB-I-FISH in detecting Ph. Our data demonstrate that FISH analysis is a rapid, reliable and sensitive technique. The comparison between BM and PB showed that PB can not be replaced by BM, even in detecting by FISH.

Chronic myeloid leukemia (CML) is a triphasic clonal myeloproliferative disease (1). It accounts for 20% of all leukemias (2). The origin of this malignant disease is hematopoietic stem cell (3-5). In about 95% of cases, the Philadelphia (Ph) chromosome is a hallmark of CML. It’s a shortened chromosome 22 that is resulted from a reciprocal translocation between long arms of chromosomes 9 and 22, t(9;22) (q34;q11). At the molecular level, as a result of the Ph translocation, t(9;22) (q34;q11), the 3´ sequences of the *Abelson (ABL)* proto-oncogene at 9q34 are joined to the 5´ sequences of the *breakpoint cluster region (BCR)* gene at 22q11, giving rise to the *BCR-ABL* hybrid or fusion gene.

*BCR-ABL* fusion gene encodes a large protein with different molecular weight whose tyrosine kinase activity is very high (4-8). This characterization signals the pathways leading to cellular proliferation, apoptosis inhibition and alterings cellular adhesion. All of these mechanisms cause the clinical manifestation of CML (9).

Although in about 95% of all CML cases, the gold standard for diagnosis and followup is conventional cytogenetics, the other 5%, including variant translocations, cryptic *BCR-ABL* rearrangements or masked Ph, are only detectable by molecular cytogenetics (5, 8, 10-12).

Fluorescence in situ hybridization (FISH) is used as a rapid and reliable molecular cytogenetic technique both in the diagnosis and subsequent monitoring of CML. On the other hand, FISH is applied to analyze both interphase and metaphase cells. Therefore, when there are no adequate metaphases, FISH is a reliable method to be used (12-15).

FISH is based on the application of DNA probes annealing to target DNA, while in, FISH analysis for CML, two probe mixes are usually applied, one for BCR and one for, which also contains a probe for the *argininosuccinate synthase (ASS)* gene (12). The BCR probe mix contains a probe 3´ of *BCR* covering a region extending 171 kb 3´, including the genes *GNAZ* and *RAB36*, while a second probe covers a 262 kb 5´ region of the gene extending 148 kb. Both are labeled in green and oriented toward that the breakpoints in the *BCR* gene (*mBCR* or *MBCR*) leads to a fusion signal. For ABL a probe contig covers a 349 kb region from the middle of the *FUB3* gene to a point of 64 kb, from the 5´ end of ABL, and it is labeled in red, whilst there is an additional red probe covering a 212 kb 3´ of *ABL*, incorporating the *ASS* gene. This additional probe is 193 kb long and spans the whole of the ASS gene. Therefore, a normal cell will show 2 red and two green signals (2R2G), while a typical translocation pattern shows 2 fusion, 1 red and 1 green signals (2F1R1G).

The aim of this study was to diagnose suspected CML patients, to monitor CML patients under imatinib therapy using cytogenetic and (FISH) techniques to analyze their bone marrow (BM) and peripheral blood (PB) samples, and finally to compare their obtained results for both specimens.

We examined 60 suspected CML patients during one-year period (2012-2013). The participants were recruited from the Hematology and Oncology Clinic of Shahid Gazi (Emam Reza) Hospital of Tabriz University of Medical Sciences, Tabriz, East Azerbaijan Province, Iran.

Sixteen females with an [average age of 45.1 years (range 20-55 years)] and forty-four males with an [average age of 45.9 years, (range 23-72 years)] were included in the study.

Among the 60 patients, 48 patients were studied at first diagnosis and the remaining 12 patients were monitored for minimal residual disease (MRD). BM and PB samples of the patients were collected transferring containers.

Chromosome analysis was performed on the cultured BM and PB cells by G-banding. PB samples were cultured in RPMI1640 medium (GIBCO, USA) with 10% fetal calf stryerum (FCS, GIBCO, USA), 1% phytohemagglutinin (PHA, GIBCO, USA), 1% L-glutamine (GIBCO, USA), and 1% penicillin-streptomycin (GIBCO, Germany) for 72 hours. BM specimens were cultured in RPMI1640 medium with 10% FSC and an addition of 1% penicillin-streptomycin for 24-72 hours.

After the samples were cultured, the colcemid (10 μg) followed by potassium chloride (KCL; 0.075 μg) was used to start harvest step on the samples. Next, the cells were fixed with a methanol and glacial acetic acid mixture (3:1). The spreading of chromosomes was performed on cold and wet slides. After slide preparation, Gbanding by trypsin using Giemsa-staining was performed according to the standard procedures. Finally, 25 metaphases were analyzed by a light microscope (Olympus, Japan).

International System for Human Cytogenetic Nomenclature (ISCN 2009) was used to analyze the chromosomes (16).

FISH was performed on metaphase cells or interphase nuclei of BM and PB samples, using a dual-color/dual-fusion *BCR/ABL* probe, for 22 and 9 chromosomes, respectively, labeled in green and red spectrums, provided by Cytocell, UK.

After slide preparation, they were immersed in 2X chloride sodium citrate (SSC) buffer (300 mmol/L sodium chloride and 30 mmol/L sodium citrate (GIBCO, USA) for two minutes at room temperature without agitation. They were then dehydrated in an ethanol series (70, 85 and 100%, respectively). Subsequently 10 μl of probe (containing both *BCR* and *ABL1* genes) was removed for test after mixing with pipette. Both the sample slide and the probe were placed on a 37˚C (+/-1˚C) hot-plate for five minutes, while 10 μl of probe mixture was spotted onto the cell sample and a cover slip with rubber solution glue was applied carefully to seal it.

Denaturation of both the slide and the probe was performed at 75˚C (+/-1˚C) for two minutes. Hybridization was carried out at 37˚C (+/-1˚C) overnight. Followed by the overnight hybridization, slides were washed in 0.4X SSC at 72˚C (+/-1˚C) for two minutes and then were immersed in 2X SSC. 0.05% Tween-20 (ROCHE, Germany) at room temperature (pH=7.0) for 30 seconds without agitation. After applying 10 μl of 4´,6-diamidino-2-phenylindole (DAPI, Cytocell, UK) antifade onto each slide, FISH signals were simultaneously evaluated on a minimum of 200 interphase nuclei, using a fluorescence microscope (Olympus BX50, Japan) equipped with specific filter sets, including DAPI, FITC, Texas Red and triple bandpass filter DAPI/ FITC/Texas Red, for viewing all fluorophores and DAPI.

The analysis and comparison of the all obtained results on PB and BM specimens using cytogenetic and FISH techniques were performed using a simple statistical analysis by determining the percentage of CML patients with Ph+ or *BCR-ABL*+.

This study in the presence of informed consent of referring patients was reviewed and approved by Islamic Azad University of Ahar, Ahar, East Azerbaijan Province, Iran, and Hematology and Oncology Research Center of Tabriz University of Medical Sciences, Tabriz, East Azerbaijan Province, Iran. The steps of the study were performed in Dr. Rahmani’s Medical Genetic Lab, Tabriz, East Azerbaijan Province, Iran and Laboratory of Hematology and Oncology Research Center of Tabriz University of Medical Sciences.

In this study, 90 specimens including 30 BM and 60 PB samples of 60 suspected CML patients were evaluated.

The present study was conducted among 16 female and 44 male patients with the average age of 52.9, whose average white blood cells (WBCs) count was 8720-385000 μl for suspected CML patients in the first diagnosis, and 3640-5410 μl for CML patients under imatinib therapy.

Out of 90 specimens used to analyze by G-banding, five specimens were not analyzable by G-banding. Therefore, FISH analysis was applied to detect *BCR-ABL* fusions. Furthermore, 30 specimens were simultaneously analyzed by both FISH and G-banding. Twenty-five samples (41.66%), out of 90 samples analyzed from 60 suspected CML patients, were Ph+ by conventional cytogenetic. Obtained results of the karyotyping on 60 PB samples (50 suspected CML patients and 10 CML patients under imatinib therapy during a 1to 5-year period) showed that twenty patients (40%), out of 50 suspected CML patients, were Ph+. None of the CML patients under imatinib therapy showed chromosomal abnormality. These findings demonstrate the complete cytogenetic response to imatinib therapy.

Obtained results of the 30 G-banded BM samples (28 suspected CML patients and two CML patients under imatinib therapy) showed that 5 BM samples, out of 28 suspected CML patients, were not evaluable by G-banding due to inaccessibility to high quality metaphase. Therefore, FISH technique was applied to detect *BCR-ABL* fusions. Fifteen patients, out of the rest 23 patients, were Ph+, and eight cases showed no chromosomal abnormality. None of the two CML patients under imatinib therapy showed chromosomal abnormality. These findings demonstrated the complete cytogenetic response to imatinib therapy.

Twenty cases out of 60 PB samples (10 suspected CML patients and 10 CML patients under imatinib therapy) were evaluated by FISH technique to detect *BCR-ABL* fusions. The *BCR-ABL* fusions were observed in only five suspected CML patients, out of 10 patients, and one CML patient under imatinib therapy (during a 5-year period) (Figs.1-3).

**Fig.1:**
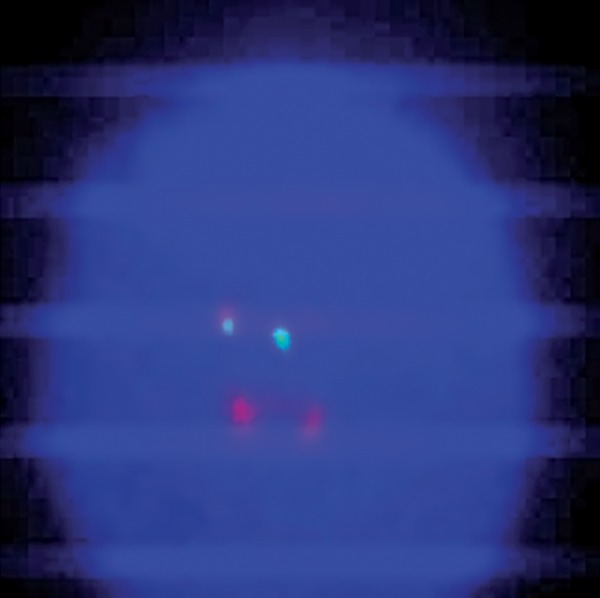
FISH signals atypical patterns of BCR-ABL fusion of PB interphase cell in CML patient (1F1G2R). FISH; Fluorescence in situ hybridization, BCR; Breakpoint cluster region, ABL; Abelson, PB; Peripheral blood and CML; Chronic myeloid leukemia.

**Fig.2:**
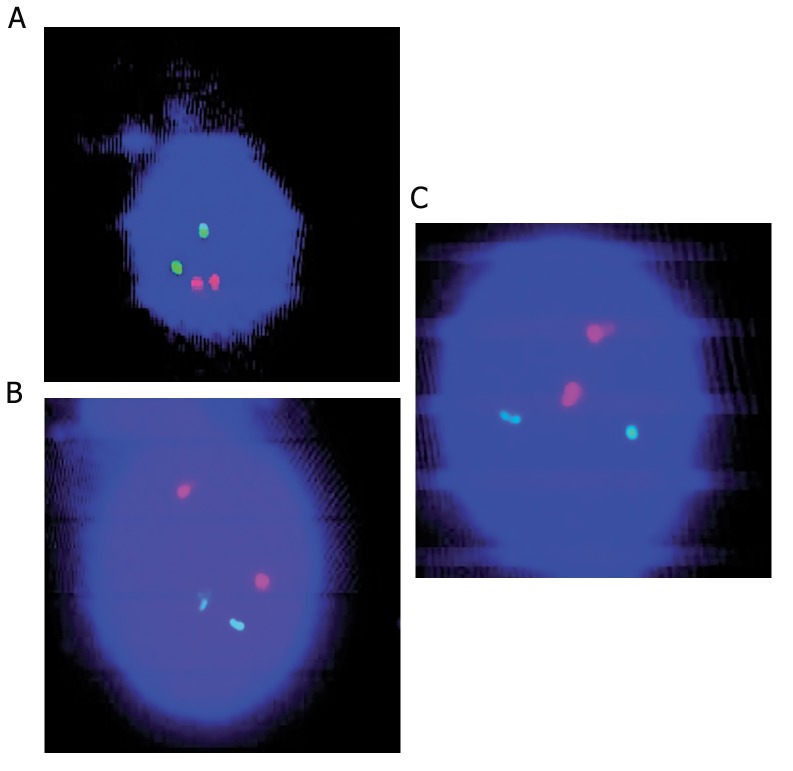
A, B and C. FISH signals patterns of normal PB interphase cell (2G2R). FISH; Fluorescence in situ hybridization and PB; Peripheral blood.

**Fig.3:**
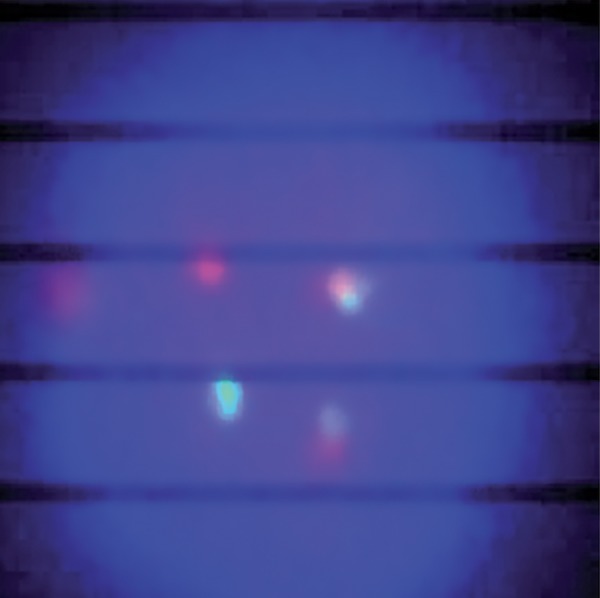
FISH signals typical patterns of BCR-ABL fusion of PB interphase cell. (2F1G1R). FISH; Fluorescence in situ hybridization, BCR; Breakpoint cluster region, ABL; Abelson and PB; Peripheral blood.

FISH analysis was utilized to detect *BCR-ABL* fusions among 15 cases out of 30 BM samples (13 suspected CML patients and two CML patients under imatinib therapy), and the results showed *BCR-ABL* fusions in 5 out of 13 patients. However, in none of the CML patients under imatinib therapy, *BCR-ABL* fusions were observed (Figs.4-7).

**Fig.4:**
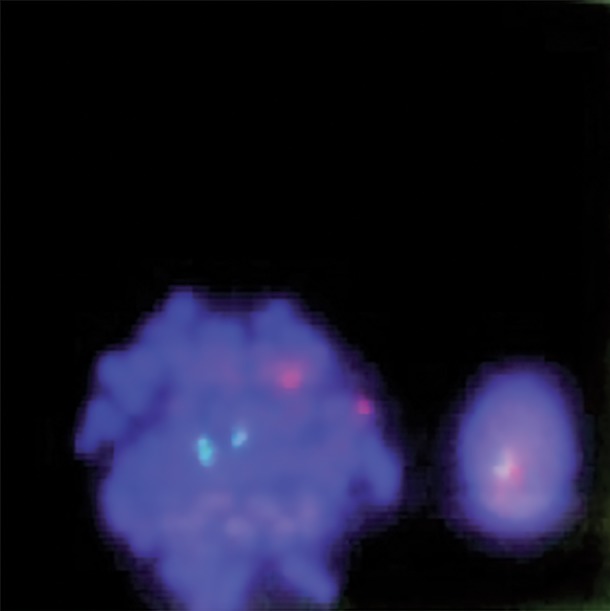
FISH signals patterns of normal BM metaphase cell (4G4R) and BM interphase cell (2G2R). FISH; Fluorescence in situ hybridization and BM; Bone marrow.

**Fig.5:**
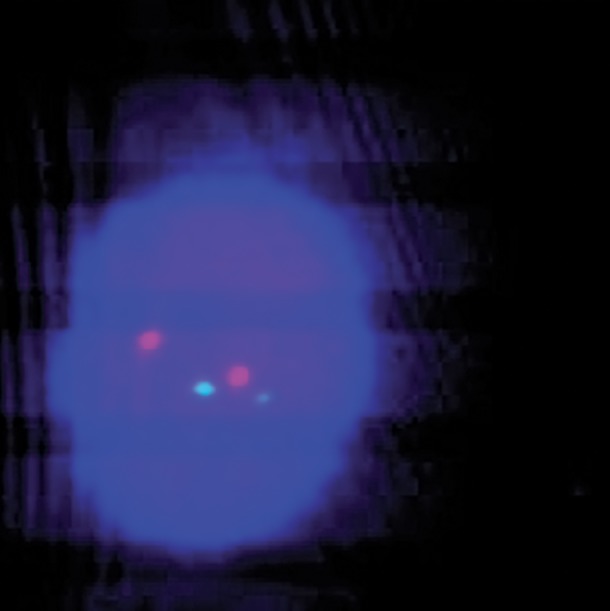
FISH signals patterns of normal BM interphase cell (2G2R). FISH; Fluorescence in situ hybridization and BM; Bone marrow.

**Fig.6:**
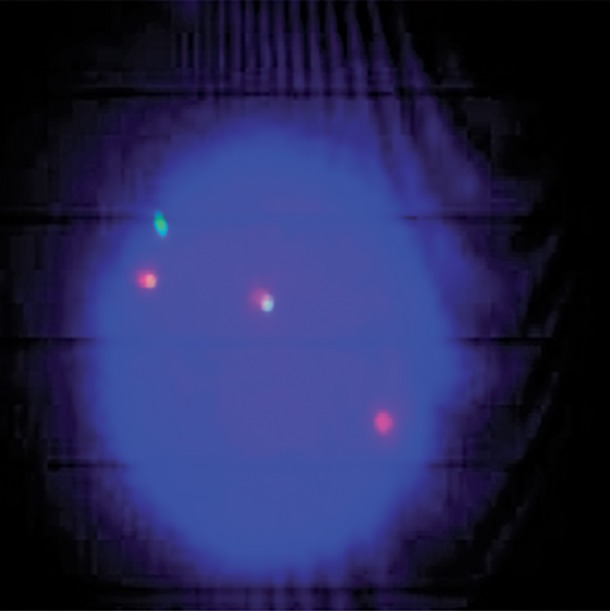
FISH signals typical patterns of BCR-ABL fusion of BM interphase cell (2F1G1R). FISH; Fluorescence in situ hybridization, BCR; Breakpoint cluster region, ABL; Abelson and BM; Bone marrow.

**Fig.7:**
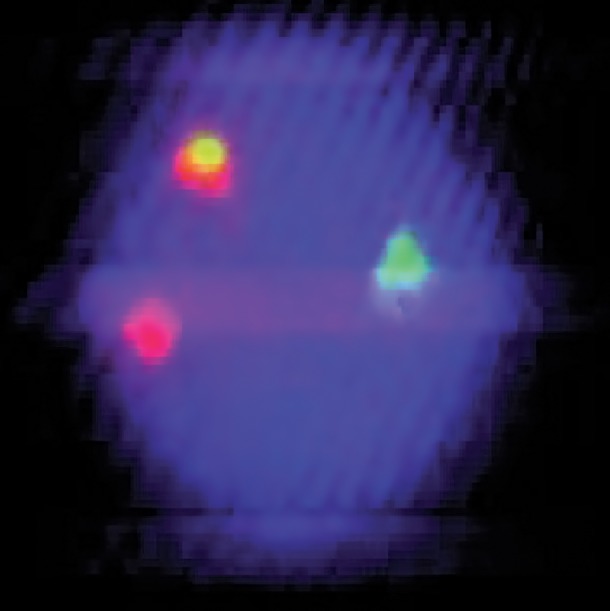
FISH signals atypical patterns of BCR-ABL fusion of BM interphase cell (1F1G1R). FISH; Fluorescence in situ hybridization, BCR; Breakpoint cluster region, ABL; Abelson and BM; Bone marrow.

Among 35 samples analyzed by FISH, atypical patterns of *BCR-ABL* gene rearrangements were only observed in two patients.

Case 1: this CML patient was under imatinib therapy during a 5-year period. In the study by dual color-dual fusion probe on the PB cells, 10% of cells had typical pattern of *BCR-ABL* fusion (2F1G1R) (Fig.3), but in five percent of them, an atypical pattern of *BCR-ABL* fusion (1F1G2R) was observed (Fig.1).

Case 2: this patient was suspected of CML and in the study by dual color-dual fusion probe on BM cells, 50 percents of cells had typical pattern of *BCR-ABL* fusion (2F1G-1R) (Fig.6). However, in the remaining 50%, atypical pattern of *BCR-ABL* fusion (1F1G1R) was observed (Fig.7).

The comparison results of G-banding and FISH technique on BM and PB samples were summarized in tables 1 and 2.

In this study, the analysis of 90 specimens belonging to 60 patients (60 PB and 30 BM samples) using conventional cytogenetic showed that 25 (41.66%) individuals of the 60 suspected CML patients were Ph+. FISH analysis was applied for five cases of BM samples and the result showed that two cases of the suspected CML patients were Ph+. These findings, which are similar to other studies, demonstrated the superiority of FISH technique when the quality or quantity of the metaphases is not good enough to be analyzed by G-banding (17, 18).

In the comparison of 25 BM and PB specimens of 60 patients using karyotyping, 15 (60%) and 10 (40%) cases were Ph+, respectively. These findings showed that BM specimens are preferable to PB specimens in the analysis by G-banding.

**Table 1:** Comparison of obtained results of CML patients under imatinib therapy on BM and PB samples by FISH and G-banding


Case	G-banding (PB)	G-banding (BM)	FISH (PB)	FISH (BM)

**1**	-Ph	-Ph	-Ph	-Ph
**2**	-Ph	-Ph	-Ph	-Ph
**3**	-Ph		+Ph	
**4**	-Ph		-Ph	
**5**	-Ph		-Ph	
**6**	-Ph		-Ph	
**7**	-Ph		-Ph	
**8**	-Ph		-Ph	
**9**	-Ph		-Ph	
**10**	-Ph		-Ph	


CML; Chronic myeloid leukemia, BM; Bone marrow, PB; Peripheral blood, FISH; Fluorescence in situ hybridization and Ph; Philadelphia.

**Table 2:** Comparison of obtained results of suspected CML patient on BM and PB samples by FISH and G-banding


Case	G-banding (PB)	G-banding (BM)	FISH (PB)	FISH (BM)

**1**	+Ph	+Ph		
**2**	+Ph	+Ph		
**3**	+Ph	+Ph		
**4**	+Ph	+Ph		
**5**	+Ph	+Ph		
**6**	-Ph	-Ph	BCR-ABL-	BCR-ABL-
**7**	-Ph	+Ph	BCR-ABL-	
**8**	-Ph	-Ph	BCR-ABL-	
**9**	-Ph	-Ph	BCR-ABL-	
**10**	-Ph	-Ph	BCR-ABL-	
**11**	+Ph	+Ph		
**12**	+Ph	+Ph		
**13**	-Ph	+Ph		
**14**	+Ph	+Ph		
**15**	+Ph	+Ph		
**16**	-Ph	+Ph		BCR-ABL+
**17**	-Ph	+Ph		BCR-ABL+
**18**	+Ph	+Ph		
**19**	-Ph	-Ph		BCR-ABL-
**20**	-Ph	-Ph		BCR-ABL-
**21**	-Ph	-Ph		BCR-ABL-
**22**	-Ph	-Ph		BCR-ABL-
**23**	-Ph	+Ph		BCR-ABL-
**24**	-Ph	no analyzable		BCR-ABL+
**25**	-Ph	no analyzable		BCR-ABL+
**26**	-Ph	no analyzable		BCR-ABL-
**27**	-Ph	no analyzable		BCR-ABL-
**28**	-Ph	no analyzable		BCR-ABL-
**29**	+Ph		BCR-ABL+	
**30**	+Ph		BCR-ABL+	
**31**	+Ph		BCR-ABL+	
**32**	+Ph		BCR-ABL+	
**33**	+Ph		BCR-ABL+	
**34**	-Ph			
**35**	-Ph			
**36**	-Ph			
**37**	+Ph			
**38**	-Ph			
**39**	+Ph			
**40**	-Ph			
**41**	+Ph			
**42**	-Ph			
**43**	-Ph			
**44**	-Ph			
**45**	-Ph			
**46**	-Ph			
**47**	-Ph			
**48**	-Ph			
**49**	+Ph			
**50**	+Ph			


CML; Chronic myeloid leukemia, BM; Bone marrow, PB; Peripheral blood, FISH; Fluorescence in situ hybridization, Ph; Philadelphia, BCR; Breakpoint cluster region and ABL; Abelson.

In comparison of FISH and karyotyping techniques on the PB and BM specimens, the obtained results were similar, but in one CML patient under imatinib therapy, FISH was able to detect *BCR-ABL* fusion in 30% of interphase blood cells, whereas in the G-banding, no Ph chromosome on the metaphases was observed. On the other hand, FISH was able to detect the atypical patterns of *BCR-ABL* fusions in this patient and the other one patient, at the time of diagnosis. These findings are in accordance with the other studies’ results, in which they showed that FISH could detect variant or masked Ph that was not detectable by conventional cytogenetic (19-22). The obtained results of comparing interphase-FISH (I-FISH) on PB and cytogenetic on BM in five patients showed that BM-cytogenetic is more reliable than PB-I-FISH in detecting Ph. However, in studies by Buno et al. (23), they have showed great ability for application of FISH to analyze PB in order to monitor response to therapy in CML patients. Furthermore in clinical examination, they have believed that cytogenetic studies on BM should be used at initial diagnosis to detect Ph+ and the other chromosome abnormalities in patients. They have also mentioned that dual fusion-FISH (D-FISH) can be used on PB, instead of BM, to assess the effectiveness of therapy (23).

The other research by Reinhold et al. (24), in which they have compared the cytogenetic and FISH methods in patients under imatinib and non-imatinib therapy and showed that I-FISH on unselected PB white cells (non neutrophils) is not proper for monitoring patients under imatinib therapy.

The findings of this research revealed that FISH is a rapid, reliable, and powerful technique by which we can detect at least 200 cells in a short time. On the other hand, by utilizing this technique, specimens do not need to be cultured and the results are obtained whithin two days. In addition, our study demonstrated the important role of FISH in detecting other atypical *BCR-ABL* fusion signals, while in the analysis by G-banding, there was no chromosomal abnormality. However, these findings need to be evaluated more by the other available FISH probes. Meanwhile, FISH is able to detect submicroscopic chromosomal rearrangements involved in CML and other leukemia diseases that are not detectable by conventional cytogenetic, and have also an important role in diagnosis of phase and prognosis of disease.

Based on the data presented, BM samples are more sensitive and reliable than PB samples; furthermore, FISH analysis on PB cannot be replaced by conventional analysis on BM. In fact, when BM specimens are evaluated by FISH, this sensitivity increases. Regarding the important roles of FISH technique in the detection of both typical and atypical signals related to leukemias and considering that these signals playing a specific role in the prognosis and severity of disease, providing different types of specific probes relevant to the involved genes in hematological malignanancies and application of this technique in medical genetic laboratory are recommended.
